# Selection of gonadotrophin surge attenuating factor phage antibodies by bioassay

**DOI:** 10.1186/1477-7827-3-49

**Published:** 2005-09-26

**Authors:** Tarja Sorsa-Leslie, Helen D Mason, William J Harris, Paul A Fowler

**Affiliations:** 1 The Department of Obstetrics & Gynaecology, University of Aberdeen, Aberdeen, AB25 2ZD, UK; 2 The Department of Molecular & Cell Biology, IMS, University of Aberdeen, Aberdeen, AB25 2ZD, UK; 3 Molecular/Cancer Biology Laboratory, Biomedicum Helsinki, University of Helsinki, POB 63 (Haartmaninkatu 8), 00014 Helsinki, Finland; 4 The Division of Basic Medical Sciences and Division of Clinical Developmental Sciences, St. George's, University of London, London, SW17 0RE, UK

## Abstract

**Background:**

We aimed to combine the generation of "artificial" antibodies with a rat pituitary bioassay as a new strategy to overcome 20 years of difficulties in the purification of gonadotrophin surge-attenuating factor (GnSAF).

**Methods:**

A synthetic single-chain antibody (Tomlinson J) phage display library was bio-panned with partially purified GnSAF produced by cultured human granulosa/luteal cells. The initial screening with a simple binding immunoassay resulted in 8 clones that were further screened using our in-vitro rat monolayer bioassay for GnSAF. Initially the antibodies were screened as pooled phage forms and subsequently as individual, soluble, single-chain antibody (scAbs) forms. Then, in order to improve the stability of the scAbs for immunopurification purposes, and to widen the range of labelled secondary antibodies available, these were engineered into full-length human immunoglobulins. The immunoglobulin with the highest affinity for GnSAF and a previously described rat anti-GnSAF polyclonal antiserum was then used to immunopurify bioactive GnSAF protein. The two purified preparations were electrophoresed on 1-D gels and on 7 cm 2-D gels (pH 4–7). The candidate GnSAF protein bands and spots were then excised for peptide mass mapping.

**Results:**

Three of the scAbs recognised GnSAF bioactivity and subsequently one clone of the purified scAb-derived immunoglobulin demonstrated high affinity for GnSAF bioactivity, also binding the molecule in such as way as to block its bioactivity. When used for repeated immunopurification cycles and then Western blot, this antibody enabled the isolation of a GnSAF-bioactive protein band at around 66 kDa. Similar results were achieved using the rat anti-GnSAF polyclonal antiserum. The main candidate molecules identified from the immunopurified material by excision of 2-D gel protein spots was human serum albumin precursor and variants.

**Conclusion:**

This study demonstrates that the combination of bioassay and phage display technologies is a powerful tool in the study of uncharacterised proteins that defy conventional approaches. In addition, we conclude that these data support suggestions that GnSAF may be structurally related to serum albumin or very tightly bound to serum albumin.

## Background

Phage display has proven to be a powerful tool for selecting proteins and peptides with specific binding properties from vast numbers of variants. Phage display is based on the simple fact that if gene fragments encoding polypeptides are fused to bacteriophage M13 coat protein genes, the protein products of these fusion genes are displayed on the surface of the filamentous phage. Studies had shown that functional antibody fragments can be expressed in the periplasmic space of *E. coli *[1,2]. In the case of antibodies, antibody fragments are displayed on the surface with the antigen-binding domains exposed to the outside environment. These phage-bearing particles can be bio-panned against immobilised antigen and those that bind can be eluted and used to infect a new *E. coli *population. This process can be repeated several times using lower concentrations of antigen each time, thus leading to significant enrichment of high affinity antigen-binding phage. The surface antibody fragments can then be sub-cloned into *E. coli *vectors that produce soluble antibodies which can be manipulated in the same way as any other recombinant protein.

Although five putative gonadotrophin surge-attenuating factor (GnSAF) amino acid sequences have been published [3-6], they have no significant homology, were derived from proteins between 12–69 kDa in mass, and have not been conclusively confirmed as GnSAF [7]. The purification of GnSAF using conventional chromatographic methods has been fraught with problems, including low concentrations of GnSAF protein in biological fluids despite high bioactivity, co-elution of GnSAF with serum albumin and the interference from large number of proteins in follicular fluid, serum and cell-conditioned medium [6,8,9]. These difficulties have hampered advances in the field. The production of specific GnSAF antibodies by conventional means has not been successful. The rat pAb reported by [6] reflects this problem, having good affinity for GnSAF bioactivity, but co-purifying inadequate amounts of protein, which are also contaminated with too many other proteins, to allow the production of homogenous GnSAF preparations.

Since conventional protein purification and polyclonal antibody strategies have failed to yield conclusive GnSAF candidate molecules, a strategy of combining phage display with our well-established rat pituitary cell bioassay for GnSAF [6,7] was developed. The rationale for this is as follows: Firstly, phage display will produce a range of antibodies to proteins even in the absence of prior purification of the proteins. Secondly, the GnSAF bioassay can be as easily used to detect the absence of GnSAF bioactivity as its presence. Thus, the bioassay could be utilised to pick out phage-derived antibodies that recognised bioactive GnSAF. Once a GnSAF-specific antibody was identified, it could be utilised in an immunopurification strategy to isolate the GnSAF molecule. The current study was therefore devised specifically to utilise the novel combination of phage display and bioassay in order to attempt to identify human ovarian GnSAF.

The rationale for the degree of effort that has been invested into GnSAF research revolves around its potential role in the negative regulation of pituitary responsiveness to GnRH [7]. GnSAF probably coordinates the LH signal with ovarian steroidogenesis and follicular development and, as such, would have considerable therapeutic and diagnostic potential for women and commercially important species.

## Methods

### GnSAF Bioactive Material and Bioassay

#### GnSAF bioassay

A critical part of this study was the GnSAF bioassay which was used to test and screen phage display library products for recognition of GnSAF bioactivity. However, the exact method employed to test these phage products varied according the stage of the study. The basic bioassay, which involves the addition of aqueous preparations for testing in a dose-response-design is described below. In the relevant sections detailing the production and testing of the phage display library products, the different approaches used to generate these aqueous preparations are outlined.

Adult female Sprague-Dawley rats (10–14 weeks old) were maintained under a constant 12-h light: 12-h dark, 22°C environment with ad libitum access to food and water. For each cell culture, 15 rats, selected at random during the estrous cycle, were killed by stunning and cervical dislocation. Dispersion and culture of the pituitary cells in serum-free defined medium (SFDM) was carried out as described [6,10]. Bioassays were carried out in quadruplicate wells: 200 μl of fresh SFDM was added, together with the treatments made up to 25 μl with SFDM. All the culture plates contained at least 12 control wells receiving SFDM only. After 24 h incubation with the test substances, the medium was collected and stored at -20°C for subsequent measurement of basal FSH as an index of inhibin bioactivity. The wells were then treated with 0.1 μM GnRH (Fertagyl: Intervet UK Ltd., Cambridge, UK) in 50 μl of SFDM. In all dishes 8 wells previously exposed to SFDM received GnRH alone while 4 wells previously exposed to SFDM received 50 μl of SFDM instead of the 50 μl of GnRH challenge. These acted as controls for the magnitude of the GnRH response. Cultures were terminated after 4 h incubation by collecting the media which was stored at -20°C for subsequent measurement of GnRH-induced LH as an index of GnSAF bioactivity (specifically reduced GnRH-induced LH, but not basal FSH secretion). The QC hFF preparations were added to each bioassay at 0, 1, 5 & 25 μl/well, in at least four wells/dose/separate culture, to act as a GnSAF quality control. Bioassays in which the QC hFF caused <30% suppression of GnRH-induced LH secretion, or in which the control GnRH response constituted <50% increase in LH, were repeated and the data discarded. Concentrations of gonadotrophins in cell-conditioned media from rat anterior pituitary cell cultures were determined using homologous rat time-resolved fluoro-immunoassay (DELFIA) for (a) FSH: with sensitivity and intra-assay and inter-assay C.V. values of 0.6 ng FSH/ml (NIDDK-rFSH-RP-2) using NIDDK-anti-rFSH-S11 and 7.1% and 11.2% respectively; (b) LH: with sensitivity and intra-assay and inter-assay C.V. values of 0.2 ng LH/ml (NIDDK-rLH-RP3) using NIDDK-anti-rLH-S11 and 5.4% and 7.9% respectively.

#### Human follicular fluid as a source of GnSAF bioactivity

All protocols employing human subjects were given Joint Ethical Committee Approval at Aberdeen and patients all gave informed consent. Follicular fluid (hFF) was aspirated from follicles ≤18 mm in diameter from 40 women undergoing routine IVF in Aberdeen and pooled and desalted as previously described [8]. Subsequently, 500 μl aliquots of the hFF pool were stored at -20°C and used as a GnSAF bioactivity quality control (QC), producing a 40–60% reduction in GnRH-induced LH at 50 μl/well, in all bioassays performed as part of the present study, as previously described [8].

#### Granulosa/luteal cell-conditioned medium (G/LCM) as a source of GnSAF bioactivity with reduced serum protein contamination

Granulosa/luteal cells (G/LC) were recovered from hFF obtained from women undergoing IVF and cultured as described by [6]. In the present study 2,500 ml of G/LC-conditioned medium (G/LCM) was processed, subjected to Dyematrex Blue A Dye reduction of serum albumin and tested to confirm GnSAF bioactivity as described [6].

### Phage Display Library Use to Generate GnSAF Bioactivity-Specific Antibodies

#### Plasmids and bacterial strains

The single-chain antibody expression vector pIMS147 was derived from the pHELP1 vector [11-14]. This vector produces soluble single-chain antibody (scAb) fragments fused via the 3' end of the variable domain with the human C_κ _(HuC_κ_) domain. This vector also contains six-histidine tag for purification by immobilised metal ion chelate affinity chromatography (IMAC). The intact immunoglobulin (IgG) molecules were obtained by using vectors VHE, VKExpress (a kind gift from A. Bradbury, Los Alamos USA) and pLNOH (a kind gift from L. Norderhaug, Oslo Norway). The modification was made by cloning the pLNOH IgG3 into VHE and adding a *Hind*III site.

#### The phage display of scAbs

500 μl of glycerol stock of the Tomlinson J Library was inoculated into 500 ml of 2 × TY broth supplemented with 1% glucose and 100 μg ampicillin/ml, and incubated, with shaking, at 37°C to an OD_600 _of 0.4 (1–2 h). KM13 helper phage [15] was added to 50 ml of the culture and the mixture incubated at 37°C, without shaking, for 30 min. Infected *E. coli *cells were pelleted, resuspended in 100 ml of 2 × TY with 0.1% glucose, 100 μg ampicillin/ml and 50 μg kanamycin/ml, and further incubated overnight, with shaking, at 30°C. Phage particles were precipitated with 20 ml polyethylene glycol in 2.5 M NaCl (20% w/v) as described previously [16]. Immunotubes (Greiner Labortechnik, Gloucestershire, UK) were coated by incubation overnight at 4°C with 4 ml of crude GnSAF preparation (10 μg total protein/ml of albumin-depleted G/LCM) in PBS (Oxoid Ltd, Hampshire, UK), washed with PBS, and blocked with 2% skimmed milk in PBS at room temperature (RT) for 2 h. Phagemid particles (approximately 1 × 10^12^) were added in 4 ml of 2% skimmed milk in PBS, and incubated at RT for 60 min on a rotating turntable, and a further 60 min without rotation. The tubes were washed 20 times with PBS containing 0.1% Tween 20 (Sigma-Aldrich Co. Ltd., Poole, Dorset, UK), and the bound phage eluted with 500 μl of trypsin-PBS (1 mg/ml) for 10 min, with rotation, at RT. Half of the eluted phage (0.25 ml) were infected into 1.75 ml of exponential phase TG1 cell culture suspension in 2 × TY broth, plated on TYE agar containing 1% glucose and 100 μg ampicillin/ml and incubated overnight at 37°C. 7 μl of 2 × TY broth containing 1% glucose, 100 μg ampicillin/ml was added to the plate and the bacteria scraped until loose. 50 μl of this cell suspension was then used to infect 50 ml of 2 × TY (1% glucose, 100 μg ampicillin/ml), incubated, with shaking, at 37°C to an OD_600 _of 0.4 and subsequently 1 ml of scraped bacteria was stored with 15% glycerol at -80°C. Selected phage were then rescued from the culture as described above. Selection (bio-panning) was repeated a further three times with reducing total protein concentrations of the immobilised GnSAF (albumin-depleted, concentrated, G/LCM) preparation (2^nd ^and 3^rd ^rounds 2.5 μg total protein/ml, 4^th ^round 1.25 μg total protein/ml).

##### Affinity selection and initial screening of anti-GnSAF antibodies

**Figure 1:**
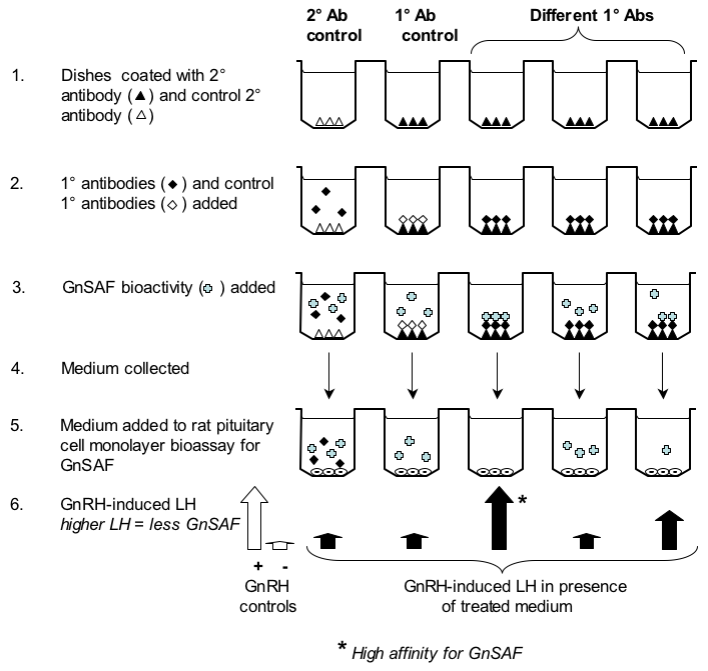
**Diagram showing the strategy to identify GnSAF-recognising scAbs**. (1) 96-well dishes were coated with secondary antibodies that would and would not bind the scAbs/IgGs and then (2) incubated with candidate anti-GnSAF and control scAbs/IgGs. When (3) GnSAF-containing QC hFF or G/LCM were added, immobilised GnSAF-recognising scAbs/IgGs bound some or all the GnSAF so that when the medium was (4) removed from the wells and added (5) to our rat pituitary monolayer bioassay, GnSAF bioactivity (6) would be reduced. Where the sequence of secondary and primary antibodies did not include the appropriate coating antibody and GnSAF-recognising scAb/IgG, GnSAF bioactivity would not be significantly reduced.

After the fourth round of bio-panning, 300 individual clones were chosen randomly. The 10 clones with the highest affinity to GnSAF, but not skimmed milk or BSA were pre-selected using an affinity enzyme-linked immunosorbent assay (ELISA) and further selection was made by sequencing them to identify complementarity-determining regions (CDRs). Final selection was based on the scAb affinity for GnSAF as determined using duplicate rat pituitary monolayer bioassays described above. The bioassay was used in the first instance to test for pooled phage clones and subsequently for individual clones which had been subcloned into a soluble vector. As detailed in Fig. 1., pooled or individual scAbs or antibodies were immobilised on dishes pre-coated with secondary antibodies and then hFF or G/LCM or culture medium alone were added. Antibodies or scAbs with affinity for GnSAF bioactivity removed GnSAF molecules from the medium. When this medium was then added to the GnSAF bioassay these GnSAF-recognising scAbs or antibodies could be detected by the reduction in GnSAF bioactivity as revealed by less marked inhibition of GnRH-induced LH release from the rat pituitary cells relative to control preparations such as hFF incubated with medium only.

##### Expression and purification of bacterial single-chain antibody fragments

A single colony of TG1 cells containing the scAb vector (pIMS147) was grown as previously described [12] and then induced with isopropyl-β-D-thiogalactosidase (IPTG) at a final concentration of 1 mM. Induction was for 4 h at 25°C, after which the periplasmic contents were released to growth media using fractionation buffer (200 mM Tris-HCl, 20% (w/v) sucrose, 1 mM EDTA, 0.5 mg lysozyme/ml, pH 7.5) [12]. The periplasmic fraction containing scAb was removed and filtered through a 0.45 μm filter (Sartorius Ltd, Epsom, Surrey, UK).

#### Purification of single-chain antibody fragments

The expressed scAbs were purified with Ni^2+^-charged fast-flow sepharose, IMAC (Amersham Biosciences UK Ltd, Little Chalfont, Bucks, UK) [14] using the hexa-histidine tail as a binding target. A Ni^2+^-charged fast-flow sepharose resin column was prepared according to the manufacturer's instructions and 2 ml of crude periplasmic fraction used to re-suspend the resin. This suspension was allowed to bind for at least 1 h at 4°C and then passed through the column 3–4 times, each time returning the suspension to the same tube. The sepharose column was washed four times with 14 ml of 100 mM NaCl in PBS, four times with 14 ml of 10 mM imidazole in PBS followed by100 mM NaCl in PBS, pH 7.9, then twice with 14 ml ice-cold PBS and allowed to settle. The column was drained and proteins eluted with 250 mM imidazole in PBS. The eluates were collected and the elution step repeated a further 3 times. Following dialysis against PBS to remove the imidazole, the eluates were assayed for expression product by capture ELISA. The eluates were placed in a Slide-A-Lyzer dialysis cassette (molecular weight cut-off 10 kDa, Pierce and Warriner Ltd, Chester, Cheshire, UK) and dialyzed against 2 litres of PBS overnight at 4°C with constant stirring, after which time the PBS was changed and dialysis was continued for a further 3 h.

#### ELISA for determination of intact IgG produced by CHO-K1 cells

96-well flat bottomed Immulon^® ^4 ELISA plates (Dynex Technologies, Ashford, Middlesex, UK) were coated either overnight at 4°C or for 1–2 h at RT with 100 μl of antibody against heavy chain (anti-human IgG, Sigma) diluted 1:1000 in PBS. The plates were washed 3 times with 200 μl of 0.05% (v/v) Tween 20 in PBS (PBS-T) and then blocked for 2 h at RT or overnight at 4°C with 200 μl of 2% skimmed milk in PBS. After washing, as described above, with PBS-T, 100 μl of the neat culture media containing IgGs was added in duplicate to all wells. The plates were incubated for 2 h at RT or 1 h at 37°C and then washed 3 times with 200 μl of PBS-T. After this, the plates were incubated for 1 h at RT with 100 μl of the anti-light chain antibody (Sigma) conjugated with horseradish peroxidase (HRP) diluted 1:1000 in PBS. A standard curve was produced by a doubling dilution series of a known concentration of purified human IgG (Sigma, 4.8 mg/ml stock). Wells containing PBS alone were included as assay blanks. After the final incubation, the plates were washed 5 times with 200 μl of PBS-T and then developed with 100 μl of 0.1 mg/ml 3,3', 5,5' tetramethylbenzidine dihydrochloride (TMB/ml) and 0.2 μl/ml of 30% (v/v) hydrogen peroxide in 0.05 mol/l citrate phosphate buffer, pH 5.0. When optimal signal intensity was obtained, the reaction was stopped by the addition of 50 μl of 1 M H_2_SO_4_. The absorbance at 450 nm was read and corrected for absorbance at 405 nm to account for non-specific absorbance by the ELISA plate on an IEMS MF plate-reader (Labsystems Affinity Sensors, Cambridge, UK). The plate-reader was controlled by Genesis software (version 2.20) for Windows™. This ELISA was repeated using concentrated, fragment-free, IgG media and a standard curve was calculated using the known IgG standard to determine the concentrations of intact IgG.

##### ScAb-derived mammalian IgGs

CHO-K1 cells were used to express scAb-derived antibodies as intact human IgGs. The cells were grown in tissue culture flasks with surface area s of 10 cm^2^, 25 cm^2 ^or 75 cm^2^, or in 96-well plates. The cultures were maintained with HAMS-F12 medium (Gibco BRL, Life Technologies Ltd., Paisley, UK) supplemented with 2 mM L-glutamine, 10% (v/v) FCS, penicillin 100 U/ml and streptomycin 100 μg/ml at 37°C in an atmosphere of 5% (v/v) CO_2 _incubator. The cells were passaged when the cultures became confluent (85–95% confluency) by splitting the cells and adding fresh media. CDRs from soluble expression plasmids (pIMS147) for two different GnSAF-recognising scAbs were amplified by PCR. Two unique cloning sites: PstI/BstEII for the heavy chain and SacI/XhoI for the light chain were then introduced. PCR products were separately sub-cloned into mammalian expression vectors for heavy and light chains (VHExpress and VKE).

#### Expression and purification of intact IgG molecules

The transfection of IgG vectors into cultured CHO-K1 cells was performed with Lipofectamine™ PLUS reagent (Gibco BRL, Life Technologies) according to manufacturer's instructions. The cells were cultured in 6-well plates, until 80% confluent, before proceeding to co-transfections. 16 μg of DNA diluted in 600 μl of serum-free Optimem media (Gibco BRL, Life Technologies) per plasmid construct (heavy and light chains) was precomplexed with 36 μl of PLUS Reagent and mixed, followed by incubation at RT for 15 min. 24 μl of Lipofectamine Reagent was diluted into 600 μl of medium (serum-free Optimem) in a second tube and mixed. Pre-complexed DNA was combined and diluted in Lipofectamine Reagent, mixed and incubated for 15 min at room temperature. Fresh serum-free Optimem media (800 μl/well) was added to the cells while the complexes were forming. DNA-PLUS-Lipofectamine Reagent complexes were added (200 μl/well) to each well, mixed into the medium gently, and incubated at 37°C at 5% CO_2_. After 3 h incubation the total volume of media was increased to 2 ml/well and the serum concentration was raised to 10%. After 24 h of transfection the media was replaced with fresh media and incubated for a further 3–4 days before collection and purification. The media, confirmed to contain the intact IgG, was concentrated 10-fold. IgG fragments were then removed by filtration through 100 kDa filter (Centricon Plus-80 PBHK Biomax, Amicon, Millipore Ltd., Watford, Hertfordshire, UK), followed by chromatographic purification with rProtein L™-Agarose (ACTIgen Ltd., Olso, Norway) according to manufacturer's instructions. An ELISA utilising antibodies against both heavy and light chains was used to monitor production and concentration.

##### Determination of scAb/IgG affinity for GnSAF bioactivity

###### (a) Binding of GnSAF bioactivity

To determine whether scAbs or scAb-derived IgGs would bind GnSAF bioactivity, *in-vitro *bioactivity-binding experiments were carried out as shown in Fig 1. In the case of the phage form of the scFvs, 96-well flat-bottomed Immulon 4 ELISA plates were coated (duplicates) either overnight at 4°C or for 2 h at RT with 200 ng of anti-M-13 antibody/well (Amersham Biosciences UK Ltd). For soluble scAb fragments in plasmid pIMS147 and for intact IgGs, produced by CHO-K1 cells, the plates were coated with 100 μl/well (1:1000 dilution) of goat anti-human kappa light chains (bound and free) antibodies (Sigma). Wells were washed three times with PBS, 0.1% Tween (200 μl/well), blocked with 2% (w/v) skimmed milk or 3% BSA in PBS for 2 h at RT or overnight at 4°C. The plates were washed with PBS-T (200 μl/well) four times, 10–50 μl per well of scAb preparation (1–10 μg) in PBS was added and the plates were then incubated for an hour at 37°C or 2 h at RT. Plates were washed again four times with 200 μl/well of PBS-T and twice with PBS before incubation with GnSAF-containing and controls preparations. The source for GnSAF for these bioassays was either QC hFF or G/LCM with GnSAF bioactivity. The GnSAF preparation was added (250 μl/well) to the immobilised scAbs/IgGs and incubated for 2 h on a rotating platform at 37°C. The fluid was recovered, duplicates combined, centrifuged at 20,000 × *g *for 10 min and the supernatant bioassayed at 0, 1, 5, 25 μl/well using duplicate rat pituitary cell monolayer bioassays.

###### (b) Blocking of GnSAF bioactivity

To determine whether scAbs or scAb-derived IgGs would block the GnSAF bioactivity, *in-vitro *bioactivity-blocking experiments were carried out as follows. A 1:1 ratio of QC hFF and scAb/IgG in PBS, or 2 μg of purified IgG with 250 μl of QC hFF, were incubated in duplicate for 2 h at 37°C on a rotating platform. The fluid was recovered, duplicates combined, centrifuged at 20,000 × *g *for 10 min and the supernatant bioassayed at 0, 1, 5, 25 μl/well using duplicate rat pituitary cell monolayer bioassays.

#### Immobilisation of ScAbs and IgGs with immunopurification supports

##### Coupling and use of scAb/IgG with CnBr-activated sepharose 4B

Cyanogen bromide activated sepharose 4 B (Pharmacia, Biotech Ltd., Knowhill, Milton Keynes, UK) was swollen for 15 min in 1 mM HCl (0.2 g/sample) and washed on a 3G sintered glass filter with the same solution (200 ml/g of dry powder). The washing solution was added in several aliquots and the supernatant was aspirated off by vacuum between successive additions. The swollen gel was then washed once with 5 ml of coupling buffer (0.1 M NaHCO_3_, 0.5 M NaCl, pH 8.3) followed by suction of the excess buffer. When the beads were dry they were added to a mixture of coupling buffer combined with scAb or IgG (1–10 μg in PBS) and rotated in an end-over-end mixer for 1–2 h at RT or overnight at 4°C. The tubes were centrifuged at 500 × *g *for 10 min and washed 5 times with 1 ml of coupling buffer. The remaining active groups were blocked by 1 M ethanolamine, pH 8.0 or 0.2 M glycine, pH 8.0 for 2 h at RT or overnight at 4°C. To remove the excess of uncoupled ligand, the adsorbent was washed alternatively with high and low pH Sodium acetate buffer (0.1 M NaAc, 1 M NaCl, pH 8.0 and pH 4.0) for 5 times. The coupled sepharose was stored in PBS at 4°C unless used immediately.

Initially, 100 μl of coupled CnBr beads were combined with 100 μl of partially purified GnSAF (2 μg of protein from albumin-depleted G/LCM), equilibrated to pH 7.4 and end-over-end rotated for 2 h at RT or at 4°C overnight. The beads were centrifuged at 500 × *g *for 5 min and the buffer was aspirated = unbound fraction. One gel volume (200 μl) of washing buffer 1 (20 mM PBS, pH 7.2) was added, mixed gently and centrifuged for 5 min at 500 × *g *and eluates collected. This wash step was repeated 3 times and eluates were pooled together with the unbound fraction. The immuno-immobilised proteins (= bound fraction) were eluted by one of two methods. The first method used 300 μl (3 gel volumes) of 0.1 M glycine, pH 2.8 and neutralised immediately after centrifugation (5 min 500 × *g*) with 120 μl 1 M Tris-HCl, pH 13. The second method used 200 μl of 2 M NaI for repeated elution steps. The eluted fractions were then combined. Proteins from a final glycine elution step were not combined with the NaI-eluted bound fractions. Both bound fractions were desalted with HiTrap desalting columns (Amersham, Pharmacia Biotech Ltd.) or microspin G-25 (Amersham, Pharmacia Biotech Ltd.) before proceeding to GnSAF bioassay at 0, 1, 5, 25 μl/well doses in two separate rat pituitary cell monolayer bioassays. Once the recovery of GnSAF bioactivity was confirmed by bioassay, this process was scaled up to immobilise 120 ng of scAb-derived IgG and perform 15 consecutive immunopurifications of GnSAF from G/LCM. The bound proteins were pooled between each immunopurification. The recovered protein was double-desalted, checked for GnSAF and inhibin bioactivities, freeze-dried and reconstituted in 200 μl of 2-D lysis buffer for gel electrophoresis.

##### Coupling and use of rpAb with Dynabeads

Two ml of rat polyclonal anti-GnSAF antiserum [6] was processed using a 5 ml HiTrap Q column according to manufacturer's instructions for the purification of rat IgG and the partially purified rat IgG desalted into 0.1 M PBS. The IgG was coupled to 300 μl of anti-rat IgG magnetic Dynabeads (Dynal Biotech Ltd, Bromborough, Wilts, UK) and then cross-linked, to enhance the stability of the immobilisation. The authors had previously (unpublished observations) determined that using 2 M NaI to elute protein bound by immobilised antibodies enabled the antibody to maintain active binding of antigen for between 10 and 15 cycles of immunopurification of G/LCM, although recovery was reduced marginally with each successive elution. Fifteen cycles of GnSAF immunopurification were then carried out. For each cycle, a fresh aliquot of 500 μl of concentrated G/LCM was incubated with the immobilised rpAb for 15 min at RT on an orbital mixer. This was designed to ensure that the binding capacity of the immobilised rpAb was exceeded every time in order to maximise the amount of GnSAF protein recovered. Unbound material was removed after each cycle by drawing the beads to the bottom of the tube magnetically and removing the medium. The beads were then washed twice with 0.2 M phosphate buffer for 3 min and the bound proteins eluted by incubation with 500 μl of 2 M NaI for 15 min at RT. The bound proteins were pooled and between each immunopurification the beads were washed twice with 0.2 M phosphate buffer for 3 min each. The recovered protein was double-desalted, checked for GnSAF and inhibin bioactivities, freeze-dried and reconstituted in 200 μl of 2-D lysis buffer (0.01 M Tris-HCl, pH 7.4, 1 mM EDTA, 8 M Urea, 0.05 M DTT, 10% (v/v) glycerol 5% (v/v) NP40, 6% (w/v) pH 3–10 Resolyte, BDH Merck Ltd., Lutterworth, Leics, UK) for gel electrophoresis.

### Other Methods

#### 1-D SDS-Page and Western Blots

SDS-PAGE was performed using Bio-Rad Mini-Protean II™ gel apparatus and SDS-polyacrylamide system supplied by Life Technologies Ltd. After separation by SDS-PAGE, the protein was electro-blotted onto a polyvinylidene fluoride (PVDF) membrane (Immobilon^®^-P, Millipore Ltd.) or onto nitrocellulose membrane (Hybond-C extra, Amersham International plc, UK). For antibody fragments containing the HuC_κ _domain, HRP-conjugated goat anti-human kappa light chain (bound and free) antibodies (Sigma) were used and for whole IgG antibodies, either HRP-conjugated goat anti-human kappa light chains (bound and free) antibody or goat anti-human IgG (whole molecule) antibody with HRP conjugate (Sigma) were used, as appropriate. The blots were developed using the ECL Western blotting detection kit (Amersham Pharmacia biotech UK Ltd.) according to manufacturer's instructions before exposing the blot onto x-ray film and developed in a Kodak M35 X-OMAT processor (Kodak Ltd., Liverpool, UK). The scAb-derived IgG and rpAb immunopurified proteins in the bound and unbound fractions were separated by 1-D SDS-PAGE.

#### 2-D gel electrophoresis

Proteins immunopurified using the immobilised scAb-derived IgG and rpAb and reconstituted in lysis buffer were analysed by 2-Dimensional gel electrophoresis (2-D gels) gels using a small format gel system [17] with immobilised pH gradient (IPG) gels for the first dimension separation. The proteins were separated in the first dimension using 7 cm, pH 4–7 IPG gel strips (Amersham-Pharmacia Biotech Ltd.). The dehydrated IPG strips were re-hydrated overnight in the IPG re-swelling buffer containing the immunopurified proteins [18]. Following their re-hydration the IPG gel strips were electrophoresed on a Multiphor II apparatus (Amersham Biotech UK Ltd.) as described in [19]. Proteins were located by staining with colloidal Coomassie brilliant blue G250.

#### Mass spectroscopic peptide mass mapping

The protein spots and bands were excised from the gels, washed, in-gel reduced, S-alkylated, and in-gel digested with sequencing-grade modified trypsin (Promega Madison, WI, USA) as described elsewhere [20,21]. An aliquot of the peptide extract produced by in-gel cleavage was passed through a GELoader tip which contained a small volume of POROS R2 sorbent (PerSeptive Biosystems, Framingham, MA, USA) as described [21]. The adsorbed peptides were washed extensively and then eluted in 0.5 μl of a saturated solution of α-cyano-4-hydroxycinnamic acid (Sigma) in 50% acetonitrile/5% formic acid. The mass spectra were acquired on a PerSeptive Biosystems Voyager-DE STR MALDI-TOF mass spectrometer operating in the reflectron-delayed extraction mode. Spectra were internally calibrated using trypsin auto-digestion products. Both the MASCOT  and the MS-Fit  database searching programs were used to search the NCBi protein database, with the masses of the tryptic peptides, to identify the proteins.

#### Statistical analysis

The *in-vitro *pituitary cell responses are expressed as percentages of the relevant control gonadotrophin concentrations secreted from blank control wells on the same culture dishes. These controls were either wells exposed to SFDM alone (basal secretion) or wells exposed to SFDM + 0.1 μM GnRH. The differences between treatment groups and dose-responses were assessed using two-way analysis of variance (ANOVA). Differences between treatments and controls were tested by Dunnet's Post Hoc Test and between treatments by the Bonferroni-Dunn Post Hoc Test. The analyses were performed using the Statview 5 programme (Abacus Concepts Inc., Berkley, CA, USA). All results are presented as means ± SEM.

#### Sequence of the study

The methods described above were combined in the following sequence: 1) production and partial purification of GnSAF bioactivity, 2) bio-panning of the Tomlinson Library, 3) identification of 300 clones by simple ELISA, 3) identification of 8 GnSAF-binding clones from the original 300 clones by bioassay, 4) subcloning of the 8 clones into soluble form, 5) selection of 3 scAbs with high affinity for GnSAF by bioassay, 6) expression of the 3 scAbs as intact human IgGs in CHO-K1 cells, 7) selection of the mammalian IgG with the highest affinity for GnSAF by bioassay, 8) immobilisation of rpAb (6) and phage-derived mammalian IgG, 9) validation of phage-derived mammalian IgG binding and blocking of GnSAF by bioassay, 10) immunopurification of GnSAF using immobilised rpAb and phage-derived mammalian IgG, 11) Western blot, 1-D, 2-D and mass spectroscopic peptide mass mapping of immunopurified GnSAF, 12) further candidate GnSAF molecules identified.

## Results

### Production of GnSAF from granulosa/luteal cells and depletion of serum proteins

**Figure 2:**
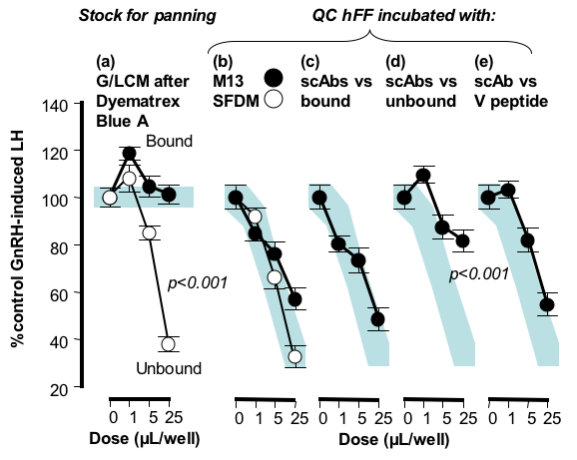
**Initial screening of pooled scAb populations**. The Dyematrex Blue A process left GnSAF bioactivity in the unbound fraction (a) and this was used to pan the phage display library. Neither anti-M13 (closed circles) nor SFDM (open circles) affected GnSAF bioactivity levels in QC hFF (b). ScAbs recognising the Dyematrex Blue A bound fraction and a non-GnSAF protein (V peptide) also had no affinity for GnSAF bioactivity (c, e) while those recognising the unbound Dyematrex Blue A fraction significantly reduced GnSAF bioactivity in QC hFF. Data represent the mean of quadruplicate determinations from 2 different rat pituitary cell culture bioassays. The shaded horizontal bar (a) indicates the mean ± SE range for control GnRH-induced LH secretion while the shaded dose-response curves (b-e) indicates the mean ± SE range for QC hFF incubated with culture medium (b, open circles). The significance value (by ANOVA) indicates the significance of reduction of GnSAF bioactivity.

BSA-free M199 conditioned by granulosa/luteal cells (G/LCM) contained significant quantities of GnSAF bioactivity with a dose of 25 μl/well reducing GnRH-induced LH secretion to <50% of control (p < 0.001). When serum albumin was depleted using Dyematrex Blue A affinity chromatography, the GnSAF activity remained in the unbound fraction, reducing GnRH-induced LH secretion to as little as 38 ± 3% of control (Fig. 2a). In contrast, the bound, serum albumin, fraction had no significant effect on GnRH-induced LH secretion (Fig. 2a).

### Selection of anti-GnSAF phage antibodies and their characterisation

The enrichment of phage antibodies from the Tomlinson J Library prior to affinity selection, and from each subsequent bio-pannning step, was indicated by the increase in the number of phage infections after each round of panning (Table I). The strategy was that selection stringency was increased by lowering the amount of antigen used to coat the immunotubes. After the 4^th ^round of bio-panning, 300 individual clones were selected randomly to produce single chain variable fragment (scFv)-phage particles. These were then individually analysed for their ability to specifically bind to crudely purified GnSAF preparation, rather than to blocking agent, by monoclonal phage ELISA. The monoclonal binding ELISA was repeated for 32 positive clones with highest affinity for the GnSAF bioactive preparations and an additional control of human serum as a binding target to eliminate any cross-reactions was added, as was the candidate GnSAF internal peptide, EPQVYVHAPC [6], for hapten finding. Since there are large numbers of proteins in serum, the degree of cross-reactivity was marked. The ELISA was repeated and the ten clones showing the strongest affinity for GnSAF bioactive preparations were selected to be sequenced and then subcloned into pIMS147 to produce phage-free antibodies. Sequencing resulted in 8 unique phage clones which were pooled and tested for recognition of GnSAF bioactivity using our *in-vitro *rat monolayer bioassay. After sub-cloning the eight clones were induced to produce soluble scFv and the binding ELISA was repeated. This confirmed that clones had retained their ability to preferentially bind GnSAF but not milk or BSA. These clones, when immobilised in 96-well plates, reduced GnSAF bioactivity *in-vitro *(data not shown, similar to data in Fig. 2.).

**Table 1:** Augmentation of phage infection rate with each round of panning against albumin-depleted G/LCM containing GnSAF bioactivity.

Panning cycle number	Amount of antigen used (μg ml^-1^)	Phage infection titre (pfu ml^-1^)
1	10.00	7.0 × 10^6^
2	2.50	8.0 × 10^7^
3	2.50	1.0 × 10^8^
4	1.25	4.7 × 10^12^

### Expression and purification of ScAbs

Unfortunately, precipitation of the scAbs was repeatedly observed after dialysing purified scAbs against PBS overnight. This could be due to aggregation or improper folding of the scAbs. To determine the concentration of scAb by HuC_κ _capture ELISA, the samples were boiled for 10 min to avoid a possible aggregation effect before addition to the ELISA plate. This enabled the overall number of scAbs in the purified preparation, but not the amount of functional scAbs, to be determined.

### Recognition of GnSAF by scAb antibodies

**Figure 3:**
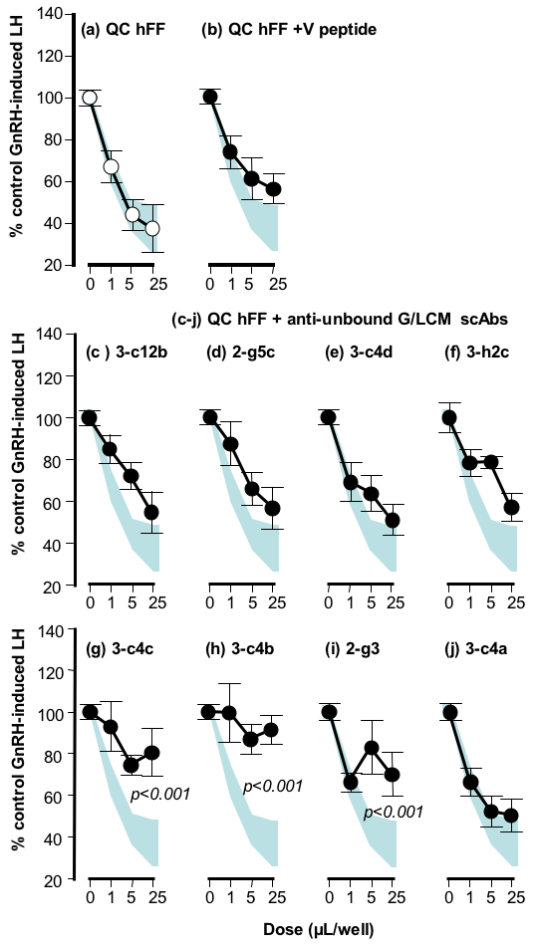
**Representative example of bioassay testing of scAb/IgG affinity for GnSAF bioactivity**. In this case antibody recognition of GnSAF bioactivity in QC hFF was tested compared with GnSAF bioactivity in QC hFF alone (a) or incubated with the non-GnSAF scAb, V peptide (b). Of the 8 individual scAbs tested, 3 showed high affinity for GnSAF, highly significantly reducing GnSAF bioactivity in QC hFF (g, h, i). Data represent the mean of quadruplicate determinations from 2 different rat pituitary cell culture bioassays. The shaded dose-response curves (a-j) indicates the mean ± SE range for QC hFF incubated with culture medium (a, open circles). The significance value (by ANOVA) indicates the significance of reduction of GnSAF bioactivity.

The pooled 8 clones were investigated for the ability to immunocapture GnSAF from QC hFF. The QC hFF incubated with culture medium alone, or with anti-M13 in the absence of the scAbs (Fig. 2b), or with the negative control a clone derived from phage library panned against a control peptide derived from a bacterial protein (V peptide, Fig. 2e), reduced GnRH-induced LH secretion to 37 ± 10%, 60 ± 2% and 55 ± 7% of control (p < 0.001 vs control). After incubation with the scAb against the non-bioactive, bound (rich in serum albumin), fraction of G/LCM following Dyematrex Blue A purification (Fig. 2c), the QC hFF retained its GnSAF bioactivity, reducing GnRH-induced LH secretion to 48 ± 7% of control, (p < 0.001 vs control). In contrast, after incubation with the scAb selected against the bioactive, unbound, fraction of G/LCM following Dyematrex Blue A purification (Fig 2d), the QC hFF had little GnSAF activity remaining (GnRH-induced LH secretion remained at 82 ± 5% of control, p > 0.05). When the clones were used to coat 96-well plates separately, 6 (Fig. 3c,d,e,f,j) had no significant effect on the GnSAF bioactivity seen in QC hFF (GnRH-induced LH secretion reduced to 37 ± 11% of control, p < 0.001, Fig. 3a) or in QC hFF incubated in V peptide-coated wells as a negative control (Fig. 3b). In contrast, GnSAF bioactivity was almost totally removed by the scAbs 3-c4c and 3-c4b and greatly reduced by the scAb 2-g3 (GnRH-induced LH secretion only reduced to 81 ± 12%, 91 ± 7% and 70 ± 10% of control respectively, Fig. 3g,h,i).

### Binding and blocking experiments with IgG forms of scAbs

Based on the bioassay data above, full length (150 kDa) immunoglobulins were produced for the scAb clones 3-c4c and 3-c4b which showed the greatest affinity for GnSAF. This was done by co-transfecting the heavy and light chain vectors (containing the scAb CDR regions) into mammalian cells CHO-K1. Transient transfections were optimised, but the transfection efficiency was far better for the light chain vector and therefore the overall intact IgG yield was relatively low. The purified IgGs were used for blocking and binding experiments. While the clone 3-c4c-derived IgG had lost its functional activity, 3-c4b-derived IgG significantly altered the hFF dose-response curves, demonstrating continued affinity for GnSAF (data not shown, dose-response changes very similar to Fig. 3h). In bioactivity-binding experiments, GnSAF bioactivity was reduced 4.6-fold compared to control IgG (transient transfections expressing an unrelated IgG recognising the pesticide hapten atrazine) while in bioactivity-blocking experiments, GnSAF bioactivity was reduced 3.4-fold compared to control (data not shown, calculated from changes in ED_50 _in dose-response curves).

### Immunopurification of GnSAF bioactivity

**Figure 4:**
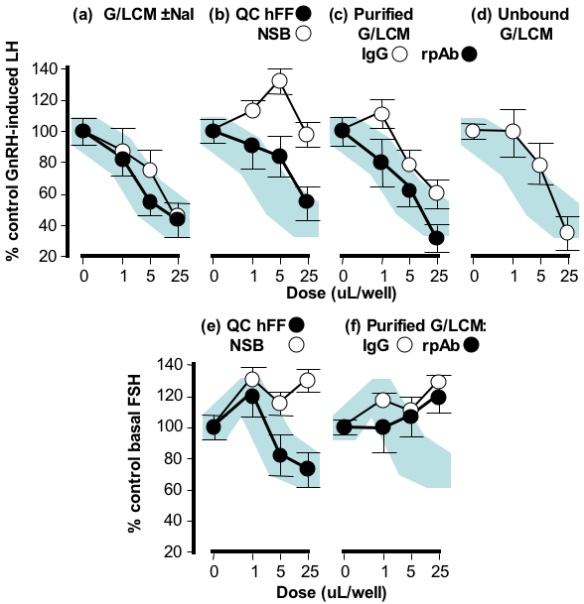
**Immunopurification of GnSAF from granulosa/luteal cell-conditioned medium (G/LCM) using immobilised phage-derived monoclonal and rat polyclonal antibodies**. (A) GnSAF bioactivity in G/LCM treated with (open circles) and without (closed circles) 2 M NaI elution buffer. GnSAF (b) and inhibin (e) bioactivities in quality control human follicular fluid (QC hFF, closed circles) but not in proteins non-specifically bound by the immunopurification media without immobilised antibody (non-specific binding: NSB, open circles). GnSAF (c) but not inhibin (f) bioactivity detected in G/LCM immunopurified using immobilised rat polyclonal (closed circles) or immobilised phage-derived antibody (3-c4b, open circles). GnSAF bioactivity remaining in excess protein not bound by immobilised phage-derived antibody (3-c4b, d). Data is from triplicate cell cultures, results are mean ± SE. The shaded dose-response curves (a-j) indicates the mean ± SE range for hFF incubated with culture medium (a, closed circles). The effects of unpurified G/LCM on basal FSH are not shown because of the high concentration of recombinant human FSH content which interferes with the rat FSH immunoassay.

**Figure 5:**
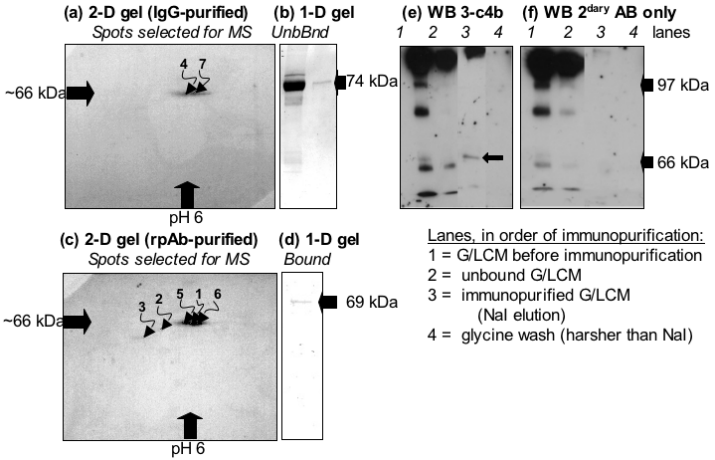
**GnSAF immunopurified from G/LCM**. Immunopurification using phage-derived antibody immobilised on protein-L agarose (a,b) or rat polyclonal antibody immobilised on anti-rat IgG-coated magnetic beads (c,d) was pooled after 15 consecutive loading and elution cycles with 2 M NaI. Proteins detected by Coomassie blue staining of (a,c) 2-D (immunopurified protein only, numbers match those in Table II) and (b, d) 1-D (Unbound = flow-through, Bound = immunopurified) gels were investigated by mass spectroscopic peptide mass mapping. Western blot of the 3-c4b-immunopurified G/LCM (e,f) with either 3-c4b and secondary antibody (e) or secondary antibody only (f) show the elution of a specifically recognised 60–70 kDa protein band (arrow) by 2 M NaI, with no further protein eluted with glycine washes.

Following small-scale experiments to ensure that the scAb-derived IgG and rpAb continued to recognise GnSAF bioactivity once immobilised, all remaining stocks of rpAb and 3-c4b-derived IgG were immobilised and used to immunopurify the maximum possible quantity of GnSAF bioactivity. These data are shown in Figs. 4 &5. An extensive panel of controls were used and these established that elution with 2 M NaI did not interfere with recovered GnSAF bioactivity (Fig. 4a). Two molar NaI processed in the same way as eluted protein also had no effect on GnRH-induced LH secretion (data not shown). The supports used for immunopurification did not bind GnSAF bioactivity (Fig. 4b) or inhibin bioactivity (Fig. 4e) in a non-specific manner. In contrast, there was marked recovery of GnSAF bioactivity (as defined by the suppression of GnRH-induced LH secretion: Fig. 4c), but not inhibin bioactivity (as defined by the suppression of basal FSH secretion: Fig. 4f), by eluted protein recognised by the immobilised rpAb and scAb-derived IgG. The recovery of GnSAF bioactivity in the unbound fraction (Fig 4d) was expected because in each cycle of immunopurification the immobilised antibodies were overloaded with GnSAF bioactivity to ensure maximal purification. When visualised by 2-D gel electrophoresis both the scAb-derived IgG (Fig. 5a) and rpAb (Fig. 5c) had produced a spread of proteins between 60 and 70 kDa, pI 5.5–6.0, the expected range for GnSAF bioactivity. Only single bands, at 74 and 69 kDa were visualised by Coomassie blue staining of 1-D gels (Fig. 5b,d). Western blotting with the scAb-derived IgG (Fig. 5e) compared with Western blot with the secondary antibody only (Fig. 5f) demonstrated specific recognition of a unique protein band at 66 kDa (Fig. 5e, lane 3).

### Candidate GnSAF molecules resulting from scab-derived IgG and rpAb immunopurification of G/LCM

The 1-D gel bands and the labelled spots in 2-D gels shown in Fig. 5 were then subjected to mass spectroscopic peptide mass mapping and the principal findings are shown in Table II. The main candidate GnSAF molecule identified was serum albumin, its precursor and variant forms. Once the human serum albumin peak was removed from the data for the 1-D rpAb-immunopurified protein, one further candidate molecule at a similar pI and kDa was revealed. The five spots visible in the 2-D gel of this material also produced positive matches with serum albumin precursor, two hypothetical proteins and other tissue-specific proteins. The keratin 10 is epidermal and therefore likely to have been due to contamination. While there was insufficient material to obtain positive identification from the IgG-immunopurified protein, the two 2-D spots again showed positive matches with serum albumin precursor and variant forms.

**Table 2:** Candidate molecules identified (NCBInr) from proteins immunopurified from G/LCM using immobilised phage-derived IgG and rat pAb.

Immuno-purification Ab	Spot/band	Database		Protein	MOWSE Score	Accession Number
					
		PI	kDa			
**rpAb**	1-D 66 kDa band	5.9	69.2	Serum albumin precursor	224,400	P02768
	Excl albumin peaks	9.08	86.8	SAD1 kinase	767	Q8TDC3
	2-D #1	6.0	56.8	PRO2619	372,800	11493459
		5.9	69.2	Serum albumin precursor	228,770	6013427
	#2	5.8	72.8	Solute carrier family 39, member 12	1,855	22749433
	#3	5.0	57.2	Keratin 10	6,260	88041
	#5	6.0	56.8	PRO2619	102,500	11493459
		5.9	69.2	Serum albumin precursor	78,270	6013427
	#6	6.0	56.8	PRO2619	2,067,000	11493459
		5.9	69.2	Serum albumin precursor	1,302,000	6013427
**Derived IgG**	1-D 74 kDa band			*Insufficient protein*		
	2-D #4	5.9	69.2	Serum albumin precursor	2.483e+11	6013427
	#7	5.9	69.4	Hypothetical protein	6.366e+11	51476390
		5.9	69.2	Serum albumin precursor	2.276e+11	6013427

## Discussion

We have successfully created anti-GnSAF antibodies with the help of phage display technology. The antigen used was partially purified GnSAF and the bio-panning was combined in a novel manner with our GnSAF rat monolayer bioassay as the selection step. This is an alteration to the normal phage display experiment where the antigen is preselected prior to phage display. It has not been possible to obtain specific GnSAF antibodies by conventional means, because the GnSAF has not yet been definitely isolated. This is highlighted by the presence of several non-GnSAF antibodies in our previously described rat anti-GnSAF serum [6]. The process of bio-panning the Tomlinson J Library against crudely purified GnSAF was successful. The increase in the number of eluted phage particles after each round of panning (Table I) caused an enrichment of clones recognised by the immobilised antigen which was further confirmed by increased signal of binding ELISAs for polyclonal phage preparations (data not shown) and recognition for GnSAF bioactivity.

After purification of the scAbs some protein aggregates or misfoldings were observed and this made it impossible to accurately quantify scAbs, also possibly reducing the number of functional scAbs in the purified preparations. The same observation has previously been made with regard to the Tomlinson J library [22]. In order to overcome this precipitation problem, the scAbs were denatured by boiling for 10 min before their addition to ELISA plates. However, this can only provide the overall number of light chains present (the secondary antibody being anti-HuC_κ_), not the number of functional scAbs. When producing scAb-derived IgGs there was no aggregation observed and accurate quantification was carried out without boiling. The coating of plastic immunoplates with antibodies relied on the immunoabsorbent nature of plates. There is always a chance that the 3-dimentional structure of antibody might be assembled in such a manner that antigen-binding site was covered or sterically hindered. When the antibody was in the phage form, an anti-M13 was used as coating agent before adding the scFv to ensure that the antigen-binding site in of the scFv was left free to be in contact with GnSAF in solution.

There have been previous reports of panning antibody libraries against "unknown" targets. For instance in one study, a mixture of proteins separated by 2-D gels was blotted onto PVDF membrane and an antibody phage display library was panned against individual spots. This process produced specific monoclonal antibodies against protein spots of interest [23]. However, this strategy produces antibodies against denatured proteins and they will not necessarily work in biological fluids where protein is in its tertiary structure. In our approach on the other hand, the protein target was in its native structure when selected by rat monolayer bioassay and thus the antibodies created this way should be functional in biological environments, for example in bioassays, and physiological systems.

The candidate GnSAF proteins identified in this study are interesting in light of a recent publication [24] suggesting that GnSAF is a C-terminal component of the human serum albumin subdomain IIIB. The main positive identification in our study was serum albumin precursor and this suggests that GnSAF could be a modified form of serum albumin. Clearly the major serum albumin forms do not have GnSAF bioactivity since the bound fraction from the Dyematrex Blue A purification step, which consists largely of serum albumin, does not have GnSAF bioactivity (Fig. 2). Some evidence of possible endocrine effects of serum albumin were published in the early 1990's, including [25], although further studies suggested that any biological activity depended upon the albumin fatty acid content rather than effects on gonadotrophin actions [26]. Nevertheless, the fact that our synthetic antibodies clearly recognised GnSAF bioactivity and also appeared to immunopurify serum albumin supports the suggestion that GnSAF may be formed by post-translational modification of serum albumin [24] or may be a much smaller molecule very tightly bound to serum albumin. What is clear however, for instance by the separation of GnSAF activity and the majority of serum albumin in bioactive preparations, is that the mature serum albumin molecule does not have GnSAF bioactivity Scaling up the production of the GnSAF-recognising IgG and immunopurifying a much larger quantity of GnSAF bioactivity than in the present study, followed by depletion of serum albumin, would be the best way of confirming or disproving this possibility. Such an approach would allow the extensive validation studies required to confirm whether a specific candidate protein is in fact GnSAF.

Although most studies indicate a 60–70 kDa size for GnSAF bioactivity [7], previously published candidates [3-6,27] are not necessarily mutually exclusive. None of the previously published GnSAF candidate sequences [3-6,27] have significant homology with the candidate proteins presented in Table 2. The only conclusion that can be drawn from this at present is that these candidates are possibly not the GnSAF molecule. [4,24] present good evidence that the serum albumin IIIB domain might be GnSAF, particularly a 12 kDa C-terminus component, possibly post-translationally modified. This is supported by our findings of serum albumin, its precursor molecule and variant PRO2619 in the immunopurified GnSAF bioactive G/LCM fractions. However, the peptide mass matches in our study are scattered across the serum albumin molecule and not limited to the amino acids after 490 of the human serum albumin molecule. Clearly this is less supportive of the findings of [4,24] and more indicative of the serum albumin being present as a contaminant or GnSAF transporter. The possible identification of SAD1 kinase once the serum albumin peaks had been excluded from the peptide mass search of the 66 kDa band of rpAb-immuniopurified G/LCM, is interesting. However, there is nothing in the limited literaure about this protein to suggest that it might be GnSAF [28]. The solute carrier molecule is a ion transporter involved in maintaining intracellular zinc concentrations [29] and therefore highly unlikely to be GnSAF. In contrast, the hypothetical protein matched to spot 7 appears to be another variant of serum albumin precursor.

Although none of the candidate GnSAF identifications were conclusive, the present study has provided the gene sequences to allow transfection to produce antibodies that recognise GnSAF bioactivity or the occupied GnSAF transporter. Scaling-up the production of the scAb-derived IgG should be linked together with antibody-based depeltion of non-GnSAF bioactive albumin to further reduce the number of proteins present in bioactive fractions. Such a strategy should ultimately lead to the final characterisation of this molecule.

A key technique in the present study was the rat pituitary cell bioassay. The use of such bioassays in the study of GnSAF was extensively reviewed by [27]. The Fowler and Danforth groups (reviewed in [7,27]) in particular undertook studies to ensure that GnSAF bioactivity as determined by bioassay was not due to steroid hormones, inhibins, activin, follistatin or some endogenous pituitary effect. Extensive *in-vivo *studies of GnSAF in women by the Messinis group (reviewed in [7,27]) also show that GnSAF requires the ovary to be present, supporting the use of G/LCM in the present study The latest such data is evidence that post-menopausal women have a more rapid response to exogenous oestradiol in terms of sensitisation of the pituitary to GnRH than would be expected [30], confirming the effect of an ovarian compound in antagonising GnRH. Nevertheless, the possibility that there are multiple proteins in G/LCM that suppress GnRH-induced LH must be considered. This point is unlikely, but will only be disproven once GnSAF is finally identified. The use of G/LCM greatly reduces the possibility of non-specific effects since no substrate for the production of steroid hormones was provided, no serum or serum albumin was used in the culture medium and the biological activity of the material has been extensively investigated [6], with similar findings seen in other species [31].

## Conclusion

We have used a novel strategy by utilising a rat monolayer bioassay in bio-panning procedure for the rapid isolation of recombinant antibody fragments from a naïve synthetic phage display library. scAbs modified to human IgG forms with the same block GnSAF bioactivity and immobilise GnSAF from biological fluids. Furthermore, we have used scAb-derived IgG to immunopurify and then Western blot a band of size 66 kDa, pI 5.5–6.0 which matches size and isoelectric behaviour suggested for GnSAF in most previous studies [7]. The repeated identification of serum albumin, precursor and variant, supports suggestions that GnSAF may be a post-translationally modified form of serum albumin, although not the mature serum albumin molecule itself. Alternatively, these identifications may be due to contamination and also suggests that GnSAF may be tightly bound to, and transported by, serum albumin. The fact that MS fingerprinting showed peptide matches across the whole serum albumin amino acid sequence introduces uncertainty as to whether GnSAF is actually part of the serum albumin domin IIIB [4,24], and supports the conclusion that GnSAF is more likely to be a separate molecule that is bound by serum albumin. Finally, this study has demonstrated how phage display and alternative screening strategies may be combined to improve protein identification strategies.

## Authors' contributions

TSL carried out the phage and antibody panning and development and drafted the manuscript.

WJH designed the phage strategy and helped draft the manuscript.

HDM helped design the study and draft the manuscript.

PAF designed the bioassay strategy, carried out the bioassays and helped draft the manuscript.

All authors read and approved the final manuscript.
